# Subgroup-Elimination Transcriptomics Identifies Signaling Proteins that Define Subclasses of TRPV1-Positive Neurons and a Novel Paracrine Circuit

**DOI:** 10.1371/journal.pone.0115731

**Published:** 2014-12-31

**Authors:** Jörg Isensee, Carsten Wenzel, Rene Buschow, Robert Weissmann, Andreas W. Kuss, Tim Hucho

**Affiliations:** 1 Department of Anesthesiology and Intensive Care Medicine, Experimental Anesthesiology and Pain Research, University Hospital of Cologne, Cologne, Germany; 2 Department for Human Molecular Genetics, Max Planck Institute for Molecular Genetics, Berlin, Germany; 3 Department of Human Genetics, University Medicine Greifswald and Interfaculty Institute of Genetics and Functional Genomics, University of Greifswald, Greifswald, Germany; University of Texas at Dallas, United States of America

## Abstract

Normal and painful stimuli are detected by specialized subgroups of peripheral sensory neurons. The understanding of the functional differences of each neuronal subgroup would be strongly enhanced by knowledge of the respective subgroup transcriptome. The separation of the subgroup of interest, however, has proven challenging as they can hardly be enriched. Instead of enriching, we now rapidly eliminated the subgroup of neurons expressing the heat-gated cation channel TRPV1 from dissociated rat sensory ganglia. Elimination was accomplished by brief treatment with TRPV1 agonists followed by the removal of compromised TRPV1(+) neurons using density centrifugation. By differential microarray and sequencing (RNA-Seq) based expression profiling we compared the transcriptome of all cells within sensory ganglia versus the same cells lacking TRPV1 expressing neurons, which revealed 240 differentially expressed genes (adj. p<0.05, fold-change>1.5). Corroborating the specificity of the approach, many of these genes have been reported to be involved in noxious heat or pain sensitization. Beyond the expected enrichment of ion channels, we found the TRPV1 transcriptome to be enriched for GPCRs and other signaling proteins involved in adenosine, calcium, and phosphatidylinositol signaling. Quantitative population analysis using a recent High Content Screening (HCS) microscopy approach identified substantial heterogeneity of expressed target proteins even within TRPV1-positive neurons. Signaling components defined distinct further subgroups within the population of TRPV1-positive neurons. Analysis of one such signaling system showed that the pain sensitizing prostaglandin PGD_2_ activates DP1 receptors expressed predominantly on TRPV1(+) neurons. In contrast, we found the PGD_2_ producing prostaglandin D synthase to be expressed exclusively in myelinated large-diameter neurons lacking TRPV1, which suggests a novel paracrine neuron-neuron communication. Thus, subgroup analysis based on the elimination rather than enrichment of the subgroup of interest revealed proteins that define subclasses of TRPV1-positive neurons and suggests a novel paracrine circuit.

## Introduction

Painful stimuli are detected by peripheral so called nociceptive neurons. They transmit sensory information from the peripheral target tissue along their neurites to neurons in the spinal cord. Further signal transmission to various brain areas results then in the experience of pain [1], [2]. Sensory neurons are classically categorized into distinct subgroups by their anatomy (thick myelinated versus thin non-myelinated fibers), their electrophysiological properties (responsiveness to various modalities and action potential properties), and/or their protein repertoire (ion channels and neuropeptides) [3]. These subgroups have been investigated intensively especially with electrophysiological approaches for their contribution to heat, cold, and/or mechanical pain [4]–[8]. The identification of components determining the functional differences between neuronal subgroups is of great interest not least for the development of mechanism-based pharmacological therapies. But, the challenge to separate subgroups of neurons from their neighboring glia and other neuronal subgroups occluded the detailed analysis of their molecular composition by e.g. transcriptome analysis. Thus it remains currently unknown, to what extent neuronal subgroups differ in their transcriptome and/or proteome and which differentially expressed proteins are important for the functionality of individual subgroups.

One nociceptive subgroup of high interest is the subgroup of TRPV1-positive neurons. TRPV1 is a non-selective cation channel, which was initially discovered by its responsiveness to noxious heat (>43°C) and to the chili pepper ingredient capsaicin [9], [10]. TRPV1 knock-out mice show insensitivity to capsaicin and impaired responses to inflammatory heat hyperalgesia [11], [12]. Specific binding sites for capsaicin have been identified by comparing avian and mammalian TRPV1 proteins [13]. Treatment of sensory neurons with capsaicin or its potent analog resiniferatoxin (RTX) causes calcium cytotoxicity that rapidly compromises and selectively deletes TRPV1(+) neurons [14]–[17]. This approach has been extensively applied to chemically ablate these neurons *in vivo* resulting in substantial improvement of various pain conditions in rodents, dogs, and monkeys [18]–[21]. Further research has demonstrated that chemical or genetic ablation of TRPV1(+) neurons predominantly abolishes heat pain, but not cold or mechanical sensitivity in mice [5], [7], [8]. These findings are currently being translated to humans in form of topical, subcutaneous, intraganglionic, or even intrathecal application of TRPV1 agonists to ameliorate various persistent pain conditions [22].

Substantial work demonstrates that TRPV1(+) neurons are heterogeneous themselves. This heterogeneity could be derived by differential activation of TRPV1 modulating signaling in cells of similar proteome. Indeed, a large number of mechanisms have been shown to dynamically regulate TRPV1 responses [23], [24]. For instance, TRPV1 directly binds to and is sensitized by protons [25], phosphoinositides (PIPs) [26], [27], calmodulin [28], scaffolding proteins [29], [30], and microtubules [31]. TRPV1 is also regulated via phosphorylation of intracellular residues by protein kinase A (PKA), protein kinase C (PKC), and Ca^2+^/calmodulin-dependent protein kinase (CaMKII) [32]–[34], or dephosphorylation by calcineurin [35]. Moreover, the quantity of active TRPV1 at the cell membrane is regulated by insertion from internal pools [36] and protein translation [37]–[38].

Alternatively, the heterogeneity of TRPV1 responses could be the result of differential expression of e.g. modulating signaling proteins. Although such a large number of molecular and cellular sensitizing mechanisms has been described, it is barely known if sensitizing signaling components are co-expressed with TRPV1 in a subgroup-specific manner. In a recent study, we could proof that indeed there is nociceptor specific expression of a signaling component. We found the regulatory PKA subunit RIIβ to show subgroup-specific expression in about 60% of sensory neurons that also express classical nociceptive subgroup markers including TRPV1 [39]. Accordingly, it needs to be addressed, if further signaling components are differentially expressed as well.

Instead of investigating them one by one, we performed a transcriptome analysis of TRPV1(+) neurons in combination with a quantitative subgroup-population analysis. In contrast to fluorescence-activated cell sorting (FACS) and/or antibody panning, which require a specific TRPV1 antibody or genetic labeling, our method is based on the chemical ablation of TRPV1-positive neurons. Accordingly, we treated dissociated dorsal root ganglia (DRG) with TRPV1-specific agonists followed by the removal of compromised TRPV1(+) neurons using density gradient centrifugation. To elucidate novel aspects of pain sensitization signaling, we then compared all dorsal root ganglia (DRG) cells including TRPV1(+) neurons with DRG cells lacking TRPV1(+) neurons by gene expression profiling and tested for subgroup-specific expression or activity of respective targets using an High Content Screening (HCS) microscopy approach established by us [39], [40].

## Results

### TRPV1 is expressed in about 44% of rat DRG neurons

**Figure 1:**
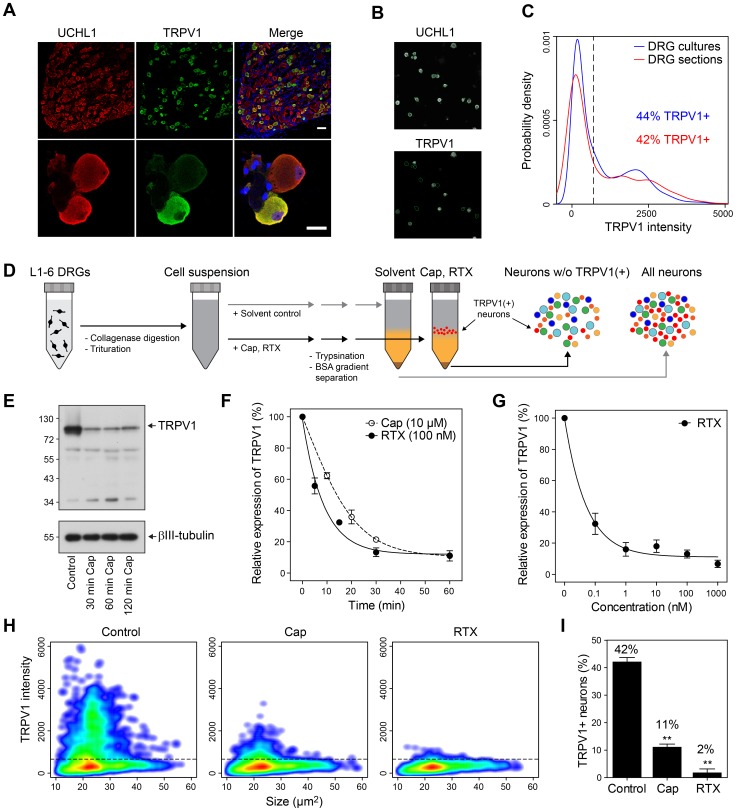
Agonist-treatment enables the selective removal of TRPV1(+) neurons. (A) Immunolabeling of the neuronal marker UCHL1 and TRPV1 in frozen DRG sections and cultured sensory neurons from rat. TRPV1 is selectively expressed in a subpopulation of sensory neurons. (B) Representative view field showing the automated image analysis to quantify TRPV1 expression in cultured sensory neurons. Green encircled objects represent sensory neurons marked by UCHL1. (C) Distribution of TRPV1 immunofluorescence intensities in frozen DRG sections (red line) and cultured sensory neurons (blue line). The scattered line indicates the threshold used to determine the number of TRPV1(+) neurons. (D) Work flow to remove TRPV1(+) neurons from freshly isolated sensory neurons. (E) Immunoblot showing the reduction of TRPV1 protein following depletion of TRPV1(+) neurons with 10 µM capsaicin for 30–120 min. (F) Time-dependent reduction of TRPV1 mRNA after removal of TRPV1(+) neurons with 10 µM capsaicin or 100 nM RTX determined by qPCR. (G) Dose-dependent reduction of TRPV1 mRNA following depletion of TRPV1(+) neurons with RTX for 30 min determined by qPCR. (H) Agonist-treated neurons were cultured overnight, immunostained for TRPV1, and analyzed by quantitative microscopy. Both agonists effectively reduced the number of TRPV1(+) neurons. Scattered lines indicate the threshold used to determine the number of TRPV1(+) neurons. (I) Quantification of TRPV1(+) neurons following treatment with 10 µM Cap or 100 nm RTX, respectively (n = 3, p<0.01, one-way ANOVA with Bonferroni's multiple comparisons test).

Reports about the size of the subgroup of TRPV1-positive neurons are varying greatly [5], [41], [42]. We now employed a highly defined quantification approach using immunocytochemical labeling followed by HCS microscopy to analyze the expression pattern of TRPV1 in DRG cultures of adult rat [39], [40]. The HCS microscopy system automatically acquires images of labeled DRG cultures in multi-well plates in up to four fluorescence channels. Neurons are identified by automated image analysis according to their expression of the neuronal marker ubiquitin carboxyl-terminal hydrolase isozyme L1 (UCHL1) in combination with object selection parameters optimized for the sphere-like geometry of neurons after short-term culture (Fig. 1A and B, and Material and Methods). TRPV1 intensities showed a broad bimodal distribution in cultured neurons and frozen sections indicating subgroup-specific but variable expression levels (Fig. 1C). To estimate the number of the TRPV1(+) neurons, we applied a fixed threshold on normalized data sets revealing 44% TRPV1(+) neurons in culture (total of 8453 neurons). This number fits with the subgroup size in DRGs *in vivo*. Analyzing frozen sections, we found 42% of DRG neurons to express TRPV1(+) (total of 1363 neurons, Fig. 1C). Corroborating the specificity of the antibody, competition experiments with the TRPV1 antigen peptide completely abolished the binding of the TRPV1 antibody (data not shown). In addition, the antibody detected a protein of the appropriate molecular weight in western blots (TRPV1 ∼95 kDa, first lane in Fig. 1E).

### Agonist-treatment enables the selective removal of TRPV1(+) neurons

Next, we set out to identify transcripts predominantly expressed in TRPV1(+) neurons. As enrichment of TRPV1-positive neurons turned out to be difficult if not impossible, we developed a method to selectively remove TRPV1(+) neurons from dissociated DRGs based on the treatment with specific TRPV1 agonists (Fig. 1D and Materials and Methods). Agonist induced opening of TRPV1 results calcium induced cytotoxicity [17]. We now found that TRPV1 agonist responsive neurons change their cellular density, which enabled us to separate them from other cells by density gradient centrifugation.

DRG neurons were treated with capsaicin (Cap, 10 µM) for 30–120 min. The treatment resulted in a substantial decrease of TRPV1 in the remaining pellet fraction determined by immunoblotting (Fig. 1E). The separated TRPV1 agonist responsive neurons could not be recollected from the supernatant. Apparently, many of the TRPV1(+) neurons disintegrated by the harsh capsaicin treatment. Thus, we set out for an inverse approach. Instead of analyzing enriched TRPV1(+) neurons directly, we compared all DRG-derived cells versus DRG cells lacking TRPV1(+) neurons.

We then performed time course experiments and quantified TRPV1 transcripts in the cell pellet by qPCR (Fig. 1F). Capsaicin as well as the more potent resiniferatoxin (RTX, 100 nM) time-dependently reduced TRPV1 mRNA. Capsaicin and RTX decreased the TRPV1 transcript level to 21±1% and 13±3% after 30 minutes of treatment, respectively (n = 3, *p*<0.001). The RTX induced TRPV1-depletion was also dose-dependent with a half-maximal effective concentrations below 0.1 nM after 30 min treatment (Fig. 1G).

To demonstrate that TRPV1(+) neurons were effectively removed, we cultured the agonist-treated cells overnight, immunostained for TRPV1, and applied HCS microscopy as described above (Fig. 1H). The number of TRPV1(+) neurons were reduced from 42±2% in the control treated sample (n = 3, 3092 neurons) to 11±1% after Cap (n = 3, p<0.01, 2808 neurons) or 2±2% after RTX treatment (n = 3, p<0.01, 1727 neurons) (Fig. 1I). The size distribution of TRPV1-negative neurons was unchanged indicating specific removal of TRPV1(+) neurons (Fig. 1H).

### Transcriptome analysis of TRPV1(+) neurons with microarrays

**Figure 2:**
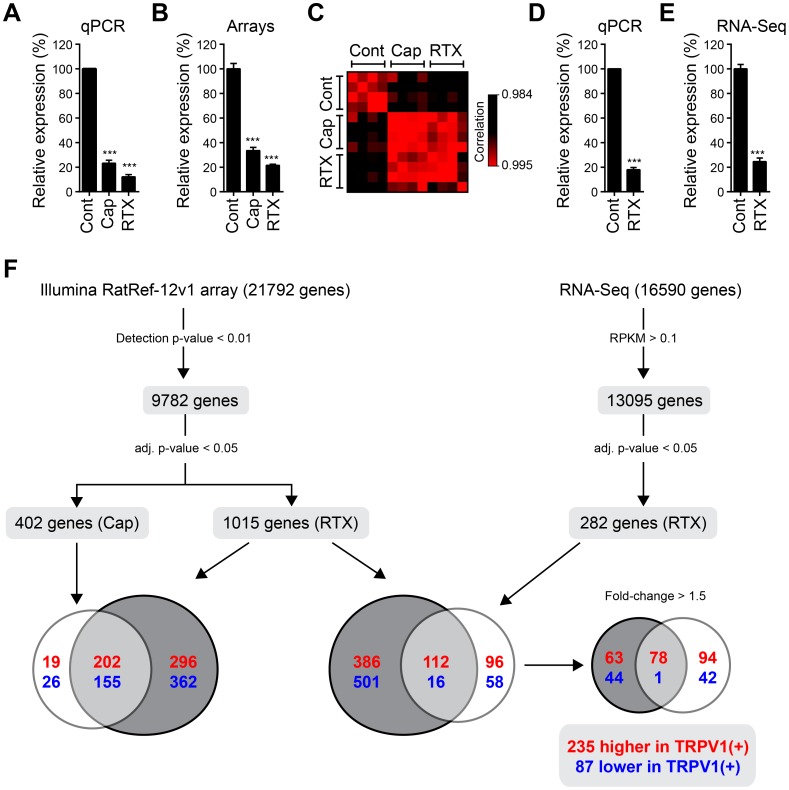
Transcriptome analysis reveals novel pain targets enriched in TRPV1(+) neurons. (A) qPCR quantification of TRPV1 mRNA levels in samples hybridized on microarrays (n = 4, p<0.001, One-way ANOVA). (B) Relative expression levels of TRPV1 mRNA determined by microarray hybridizations (n = 4, p<0.001, One-way ANOVA with Bonferroni's multiple comparisons test). (C) Correlation matrix of the expression level of all genes (normalized fluorescence intensities) detected using micorarrays. Clustering of overall gene expression is visible between RTX and capsaicin treated samples. (D) qPCR quantification of TRPV1 mRNA levels in samples used for RNA-Seq (n = 3, p<0.001, paired two-tailed t-test). (E) Relative expression levels of TRPV1 mRNA determined by RNA-Seq (n = 3, p<0.001, One-way ANOVA). (F) Overview of the number of target transcripts identified by microarray hybridizations and RNA-Seq. Raw fluorescence intensities of microarrays were background corrected, log2 transformed, normalized, and filtered for expressed genes (Illumina detection p-value <0.01 in at least one of the samples, see Material and Methods sections for details). Sequencing was performed using a 5500xl SOLiD System resulting in 664 million reads in total (see Material and Methods sections for details). The reads were filtered to remove ribosomal RNA, tRNAs, and vector sequences. The remaining reads mapped to 16590 genes of the reference genome (rn5). Read counts were transformed to RPKM values (Reads per kilo base per million), normalized, and filtered to remove weakly expressed transcripts (RPKM>0.1). P-values of differentially expressed genes identified with both methods were adjusted for multiple testing with Benjamini and Hochberg's method, adjusted p-values <0.05 were considered significant.

The selective removal of TRPV1(+) neurons as described above allowed us to perform differential transcriptome analysis with Illumina's microarray platform. To gain statistical power and account for biological variation, we performed four biological replicate experiments. For each experiment, the suspension of freshly dissociated DRG neurons of a single rat was separated into three parts and treated for 30 min with DMSO (0.1%), capsaicin (10 µM), or RTX (100 nM), respectively. To verify the successful removal of TRPV1(+) neurons, we quantified TRPV1 mRNA levels by qPCR. As before, capsaicin and RTX reduced TRPV1 mRNA to 23±1% (−4.3-fold, n = 4, p<0.0005) or 12±1% (−8.3-fold, n = 4, p<0.0005), respectively (Fig. 2A).

We then hybridized the samples on Illumina RatRef-12v1 microarrays (22519 probes for 21792 genes). The analysis of normalized signal intensities revealed a 3.0-fold or 4.6-fold reduction of TRPV1 expression levels in capsaicin- or RTX-treated samples (Fig. 2B). In addition, we observed a strong correlation between biological replicates of the same condition indicating a reproducible setup (Fig. 2C).

Filtering for expressed genes (detection p-value <0.01) resulted in 9782 genes (10131 probes) representing 45% of genes on the array (Fig. 2D, S1 Table). Of those we found 402 and 1015 being differentially expressed in the capsaicin- and RTX-treated conditions, respectively (Benjamini and Hochberg adjusted p-value <0.05, S2 Table). The higher number of significantly regulated genes in RTX-treated samples reflects the stronger effect of RTX. The genes identified using RTX included 89% of genes found with capsaicin indicating a similar effect of both compounds. Microarray data were deposited at GEO database (GSE59727).

### Transcriptome analysis of TRPV1(+) neurons with RNA-Seq

We also applied RNA sequencing (RNA-seq), which provides advantages over microarrays such as a larger dynamic range of detection, the number of reads mapping to a gene is proportional to the transcript abundance, and it reveals alternatively spliced transcript variants [43].

Since RNA-Seq required larger amounts of RNA, we performed three replicate experiments with pooled RNA from DRG neurons of three rats per experiment. Dissociated neurons were split up in two parts, treated with solvent DMSO (0.1%) or RTX (100 nM), followed by gradient centrifugation. Quantification of TRPV1 mRNA levels by qPCR verified a reduction to 18±2% (−5.6-fold, n = 3, *p*<0.0005) (Fig. 2C). Sequencing as a pool of barcoded samples on three lanes of a SOLiD 5500xl flowchip resulted in 664 million reads in total mapping to 16590 unique genes (Fig. 2F). To provide a comparable quantitative approximation of transcript abundance, read counts were transformed to RPKM values (Reads per kilo base per million) [44]. As an approximation, <1 RPKM corresponds to weak expression, 10–100 RPKM to moderate expression, and>100 FPKM to high expression. Filtering for genes with expression levels>0.1 RPKM to exclude weakly expressed genes resulted in 13095 genes (S3 Table). This is comparable to recent RNA-Seq data on DRG neurons of mice [45]. As expected, we found TRPV1 with an expression level of 104 RPKM indicating high expression in sensory neurons. Testing for statistically significant differences (adjusted p-value <0.05) revealed 282 genes of which 208 were higher and 74 were lower expressed in TRPV1(+) neurons, respectively (S4 Table). TRPV1 ranked at position eleven with a fold-change of 4.1 (Fig. 2E).

### Microarray and RNA-Seq substantially overlap

**Figure 3:**
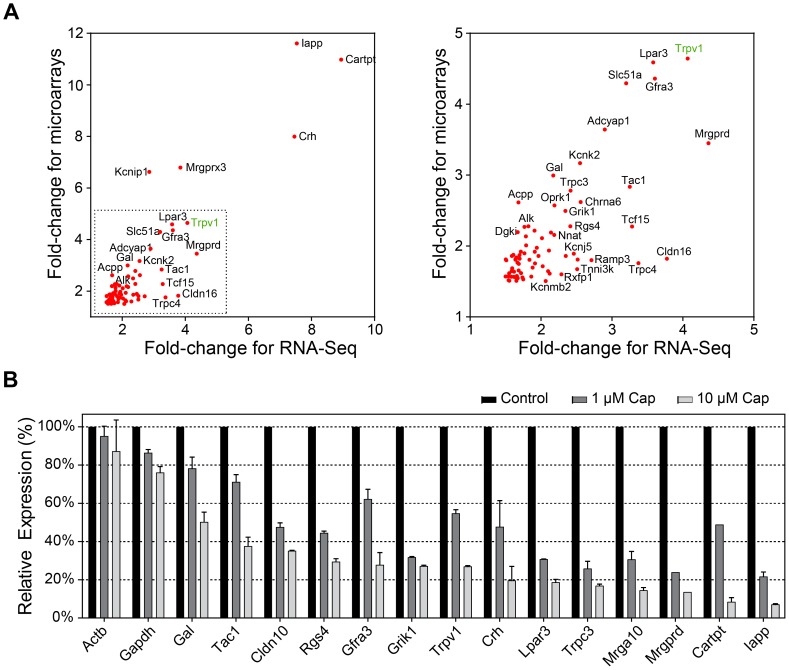
Validation of transcriptome data. (A) Correlation of fold-changes obtained by microarray hybridizations and RNA-Seq (Spearmans ρ = 0.66, p<0.0001). (B) qPCR validation of 14 transcripts identified as differentially expressed by microarray hybridizations or RNA-Seq. The depletion of TRPV1(+) neurons was performed with capsaicin (1 and 10 µM) for 30 min. The reduction of all target transcripts is dose-dependent.

We then merged the microarray and sequencing results obtained using RTX and selected candidates with p-values <0.05 and fold-changes>1.5 using one of the two approaches (S5 Table). Of the remaining candidate genes, 235 were higher and 87 were lower expressed in TRPV1(+) neurons, respectively (Fig. 2F). Of note, the overlap of genes enriched in TRPV1(+) neurons detected with both methods was substantial (34%). In addition, the fold-changes of these candidates correlated significantly (Spearmans ρ = 0.66, *p*<0.0001, Fig. 3A). A list with the top 50 transcripts found with higher expression levels in TRPV1(+) neurons is shown in Table 1. The larger number of genes detected by microarray analysis is likely caused by increased statistical power due to a larger number of replicates (n = 4 for microarrays vs. n = 3 for RNA-Seq). Further disparities between the platforms include wrong microarrays probes or expression of splicing variants lacking the complementary probe sequences.

**Table 1:** Top 50 transcripts found with higher expression levels in TRPV1(+) neurons by microarrays and/or RNA-Seq (adj. p<0.05, fold-change>1.5, RPKM>0.5).

Symbol	Description	Gene ID	RPKM	Fold-changes		Adj. p-values	
				Seq	Array	Seq	Array
**Iapp**	Islet amyloid polypeptide	24476	19.5	7.5	11.6	4.6E-26	6.1E-07
**Cartpt**	Cocaine- and amphetamine-regulated transcript	29131	9.9	8.9	11.0	3.7E-38	1.9E-07
**Crh**	Corticotropin-releasing hormone	81648	3.8	7.5	8.0	1.0E-23	4.8E-05
**Mrgprx3**	Mas-related G-protein coupled receptor member A	252960	20.8	3.8	6.8	1.6E-25	1.0E-08
**Hal**	Histidine ammonia-lyase	29301	1.1	6.7		6.2E-13	
**Kcnip1**	Kv channel-interacting protein 1	65023	31.5	2.9	6.6	2.8E-18	2.7E-04
**Ptgdr**	Prostaglandin D2 receptor	498475	3.1	5.0		2.0E-13	
**Trpv1**	Transient receptor potential cation channel subfamily V member 1	83810	64.3	4.1	4.6	1.4E-35	3.4E-09
**Nos1**	Nitric oxide synthase, brain	24598	1.8	4.6		3.3E-21	
**Slc16a12**	Monocarboxylate transporter 12	309525	8.9	4.6		7.4E-28	
**Lpar3**	Lysophosphatidic acid receptor 3	66025	10.6	3.6	4.6	1.4E-22	4.6E-05
**Mrgprd**	Mas-related G-protein coupled receptor member D	293648	27.8	4.4	3.4	3.2E-31	6.7E-05
**Gfra3**	GDNF family receptor alpha-3 precursor	84422	31.3	3.6	4.4	8.3E-28	2.4E-06
**Slc51a**	Solute carrier family 51, alpha subunit	303879			4.3		6.2E-08
**Lmx1a**	LIM homeobox transcription factor 1 alpha	289201	1.4	3.8		3.9E-11	
**Adcyap1**	Pituitary adenylate cyclase-activating polypeptide	24166	33.6	2.9	3.6	4.7E-18	5.3E-08
**Trpc4**	Short transient receptor potential channel 4 isoform beta	84494	0.9	3.4	1.8	2.1E-06	2.7E-03
**Ascl4**	Protein Ascl4	299687			3.4		4.8E-05
**Pnmal1**	PNMA-like protein 1	361515	1.1	3.4		1.1E-04	
**Vwa5a**	von Willebrand factor A domain-containing protein 5A	301097	2.6	3.3		1.0E-15	
**Tcf15**	Transcription factor 15	296272	0.9	3.3	2.3	1.9E-08	1.2E-04
**Tac1**	Protachykinin-1	24806	256.4	3.3	2.8	2.9E-26	1.8E-06
**Ewsr1**	RNA-binding protein EWS	100912481	1.2	3.2	1.0	1.0E-04	9.4E-01
**Slc51a**	Organic solute transporter subunit alpha	303879	7.2	3.2		5.0E-14	
**Zic5**	Zinc finger protein ZIC 5	361095	9.0	3.2		3.3E-17	
**Kcnk2**	Potassium channel subfamily K member 2 isoform 2	170899	7.3	2.5	3.2	4.0E-12	2.6E-05
**Amdhd1**	Probable imidazolonepropionase	299735	4.4	3.0		3.3E-09	
**LOC257642**	rRNA promoter binding protein	257642	31.1	3.0		3.6E-21	
**Gal**	Galanin	29141	16.3	2.2	3.0	2.6E-06	2.1E-05
**Trpc3**	short transient receptor potential channel 3	60395	12.3	2.4	2.8	2.4E-12	2.1E-05
**Rgs14**	Regulator of G-protein signaling 14	114705	1.4	2.7	1.1	9.2E-06	3.0E-01
**Cd72**	B-cell differentiation antigen CD72	313498	3.0	2.7	1.9	8.5E-06	3.2E-01
**Ramp3**	Receptor activity-modifying protein 3	56820	20.6	2.7	1.8	2.8E-14	1.8E-02
**Chrna6**	neuronal acetylcholine receptor subunit alpha-6	81721	34.1	2.6	2.6	7.4E-15	1.2E-05
**Acpp**	Prostatic acid phosphatase	56780	82.9	1.7	2.6	1.1E-04	2.9E-06
**Oprk1**	Kappa-type opioid receptor	29335	5.3	2.2	2.6	9.0E-05	7.0E-04
**Prdm8**	Uncharacterized protein	305198			2.6		6.1E-07
**Cpne5**	copine-5	309650	1.7	2.5	1.8	2.5E-07	3.6E-03
**Tnni3k**	serine/threonine-protein kinase TNNI3K	295531	1.1	2.5	1.7	4.3E-04	4.7E-03
**Grik1**	glutamate receptor, ionotropic kainate 1 isoform 3	29559	75.1	2.3	2.5	1.3E-13	5.3E-07
**Ipcef1**	Interactor protein for cytohesin exchange factors 1	361474	1.8	2.5		2.3E-03	
**Kcnj5**	G protein-activated inward rectifier potassium channel 4	29713	6.6	2.5	1.9	6.5E-07	3.7E-02
**Spata32**	spermatogenesis associated 32	287747	1.6	2.4		2.2E-02	
**Rgs4**	Regulator of G-protein signaling 4	29480	561.0	2.4	2.3	5.2E-15	5.1E-07
**Sepw1**	selenoprotein W		3.5	2.4	1.1	6.3E-03	4.6E-01
**Krt75**	keratin 75	300247	1.6	2.3	1.9	2.1E-03	3.8E-02
**Pfn2**	Profilin-2	100909840	1.1	2.3	0.8	1.4E-04	4.0E-02
**Foxs1**	forkhead box protein S1	311547	2.7	2.3	1.0	1.4E-04	9.9E-01
**Inadl**	InaD-like (Drosophila)	140581			2.3		5.5E-05
**Galr2**	Galanin receptor type 2	100910349	19.5		2.3		6.7E-06

### The reduction of differentially expressed transcripts is dose-dependent

To confirm the results obtained by transcriptome analysis, we selected a set of 14 target transcripts according to fold-change, RPKM-value, and relevance in the context of nociceptor functionality. These targets were validated by qPCR in independent samples treated with two different doses of capsaicin (1 µM and 10 µM) to achieve a half-maximal and full elimination of TRPV1(+) neurons. We found a dose-dependent reduction of transcript levels for all 14 targets (Fig. 3B). In contrast, relative expression levels of the house keeping genes Actb and Gapdh were not significantly affected.

### TRPV1(+) neurons are enriched for heat pain relevant genes

To demonstrate the functional relevance of our dataset, we examined whether our candidate gene list includes genes known to be relevant for pain. For this purpose, we compared our top 235 candidates (Fig. 2F and S5 Table) with all 367 entries in the Pain Networks database [46]. We found a significant overlap of 27 genes (p = 6.5e-14, modified Fisher's exact test), most of which are well-studied targets for pain (S6 Table). We also observed an overlap of 29 genes (p = 2.5e-16) when comparing our gene set with all 340 entries in the Pain Genes database [47], which lists all genes that induce a significant behavioral pain phenotype if knocked out in mice (S6 Table). Moreover, we determined the intersection of our gene set with the results of a recent genome-wide Drosophila screen for genes involved in heat nociception identifying 580 candidate genes mapping to the rat genome [48]. Six rat orthologs of these genes were also present in our gene set (Galr2, Kcnip1, Kcnip2, Kcnip4, Slc5a7, Trpv1, p = 0.56).

### TRPV1(+) neurons are enriched for potassium channels

To deeper understand the biological meaning of the dataset, we analyzed the combined dataset of TRPV1 associated genes (fold-change >1.5, p<0.05, 240 genes) for overrepresentation of Gene Ontology (GO) terms, Kegg pathways, and Interpro protein domains using DAVID [49]. Several GO terms for general neuronal processes such synapse, dendrite, and axon terminus were enriched in the dataset (S7 Table). In addition, terms such as “response to pain” and “sensory perception of pain” indicated enrichment of pain relevant genes and included well-known pain targets (Calca, Grik1, Trpv1, P2rx3, Tac1, Iapp, Npy1r). Apparently most of the top GO categories were related to ion channel activity (32 genes in total) with superior emphasis on cation channel activity (27 genes). This gene cluster contained 11 potassium channel subunits of the voltage-gated (Kcna3, Kcnc4, Kcne3, Kcnf1, Kcng1, Kcnh6), two-pore (Kcnk2, Kcnk18), inwardly rectifying (Kcnj5), calcium-activated (Kcnmb2) and sodium-activated subfamilies (Kcnt1) as well as three Kv channel-interacting proteins (Kcnip1, Kcnip2, Kcnip4). Moreover, calcium channels (Cacna1e, Cacna2d1), Trp channels (Trpc3, Trpc4, Trpv1), the voltage-gated sodium channel NaV1.9 (Scn11a), as well as ionotropic glutamate (Grik1, Grik3) and nicotinic acetylcholine receptors (Chrne, Chrna6) were found with increased expression in TRPV1(+) neurons.

### TRPV1(+) neurons are enriched for GPCRs and associated signaling components

Genes associated with G-protein coupled receptor signaling pathways were significantly enriched in TRPV1(+) neurons (S7 Table). These include orphan GPCRs (Gpr27, Gpr35, Gpr68), phospholipid and prostanoid receptors (Lpar3, Ptgdr, Ptgdrl, Ptger3, Ptgir), Mas-related GPCRs (Mrgprd, Mrgprx3), peptide receptors (Galr2, Npy1r, Npy5r, Rxfp1, Sstr1), dopamine receptors (Drd2), and opioid receptors (Oprk1). Several GPCR ligands such as pituitary adenylate cyclase-activating polypeptide (Adcyap1), calcitonin-related polypeptide α (Calca), cocaine-and amphetamine-regulated transcript protein (Cartpt), corticotropin-releasing hormone (Crh), galanin (Gal), and amylin (Iapp) were expressed at higher levels in TRPV1(+) neurons. The GPCR-related GO categories also contained G-proteins (Gnaq, Gna14), regulators of G protein signaling and GPCR kinases (Rgs4, Rgs7, Rgs14, Ramp3, Adrbk2), and GPCR signaling proteins (e.g. adenylate cyclase V, Adcy5).

### Signaling proteins define distinct subgroups of TRPV1-positive neurons

**Figure 4:**
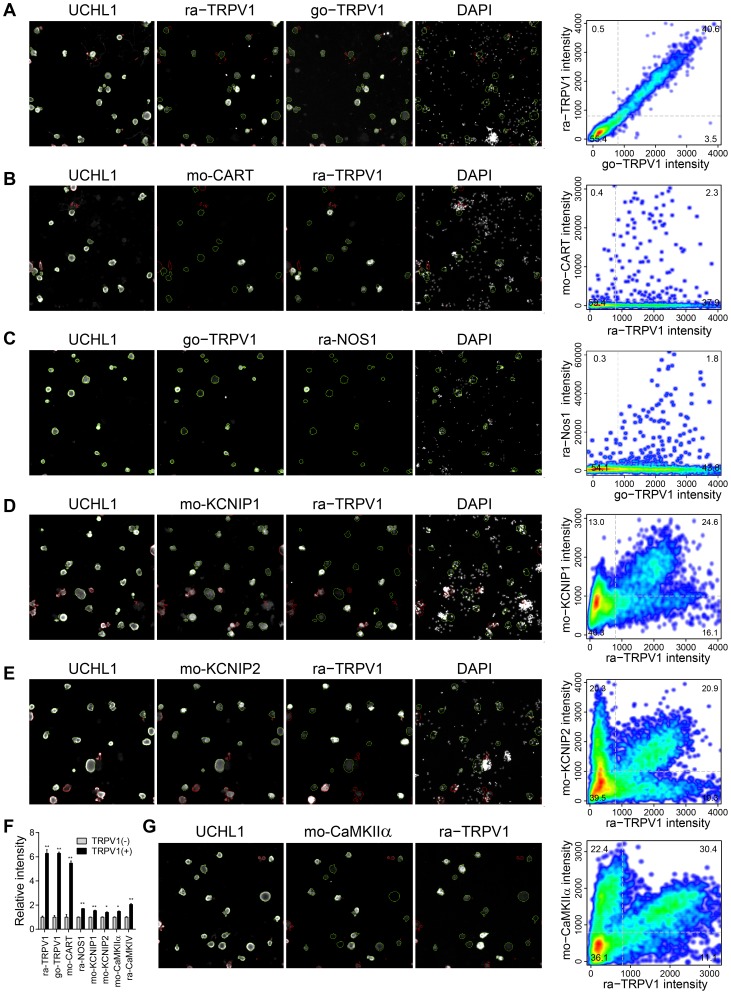
Validation of the transcriptome data by single cell based quantitative High Content Screening (HCS) microscopy focusing on selected signaling-relevant proteins. (A) Triple staining of the neuronal marker UCHL1 and two different TRPV1 antibodies derived from rabbit and goat, respectively, to facilitate the analysis of various targets. The staining intensities obtained with both TRPV1 antibodies correlated significantly (Spearmans ρ = 0.96, p<2.2e-16). (B-E, G) Co-labeling of TRPV1 and CART (B), Nos1 (C), KChIP1 (D), KChIP2 (E), and CaMKIIα (G). Plots of respective controls are shown in S1 Fig. (F) Average fluorescence intensities of TRPV1 and the indicated targets in TRPV1-negative (grey) and -positive (black) neurons. Signal intensities of all analyzed targets were significantly higher within the TRPV1(+) population (n = 3 with>3000 analyzed neurons per experiment, paired two-tailed t-tests).

Subsequently, we analyzed whether the enrichment of target transcripts in TRPV1(+) neurons is also reflected at the protein and cellular level. We focused on proteins involved in exemplary aspects of signaling such as the neuropeptide cocaine- and amphetamine-regulated transcript protein (CART), the intracellular signaling enzymes neuronal nitric oxide synthase (Nos1) and Ca2+/calmodulin-dependent protein kinases (CaMKs), as well as Kv channel-modulating proteins (KChIPs). High fold-changes detected by the transcriptome analysis of this heterogeneous cell system can have various reasons including (I) the target is expressed exclusively in the same subpopulation as TRPV1, (II) the target is selectively expressed in a subpopulation of TRPV1(+) neurons, or (III) the target is expressed in all neurons, but with higher levels in TRPV1(+) neurons. We therefore correlated the cellular expression pattern of selected targets proteins with TRPV1 using quantitative HCS microscopy. The targets were selected based on their fold-change, RPKM value, and availability of specific monoclonal antibodies. To facilitate the triple staining of UCHL1, TRPV1, and various targets, we established a second TRPV1 antibody derived from goat (go-TRPV1). The staining intensities derived with goat and rabbit TRPV1 antibodies correlated significantly (Spearmans ρ = 0.96, p<2.2E-16, Fig. 4A, B).

The neuropeptide CART was found being differentially expressed using microarrays (Cartpt, 11.0-fold) and RNA-Seq (8.9-fold, RPKM = 18) (Table 1). The reduction of CART mRNA was also dose-dependent shown by qPCR (Fig. 3B). The receptor of CART is yet unknown, but it has been suggested to potentiate spinal pain transmission [50]. A previous study in mice reported that ∼50% of the peptidergic nerve terminals in lamina I of the dorsal horn also contain CART [51]. We found high CART expression levels in a small subpopulation of sensory neurons by HCS microscopy (2.9±0.1%, n = 3, total of 11091 neurons, Fig. 4C), most of which coexpressed TRPV1 (79±4%). The immunoreactivity for CART was also significantly higher in TRPV1(+) neurons (5.4-fold, p<0.0001, Fig. 4H) verifying our transcriptome data at protein level.

We then analyzed the expression pattern of the neuronal nitric oxide synthase (Nos1, 4.6-fold, RPKM = 3). Nitric oxide has a well-established role for the nociceptive signal transmission in the spinal cord. Several animal studies have shown that inhibition of NO reduces inflammatory and neuropathic pain [52]. Similar as for CART, we observed high Nos1 expression in a small subgroup of sensory neurons (2.0±0.2%, n = 3, total of 18397 neurons, Fig. 4D). Most of these neurons were TRPV1(+) (86±1%) and also the signal intensity was significantly higher in TRPV1(+) neurons (1.7-fold, p<0.001, Fig. 4H).

We noticed enriched Ca2+/calmodulin-dependent protein kinase activity in TRPV1(+) neurons by GO analysis. CaMKs are downstream effectors of calcium and phosphorylate substrates involved in exocytosis, transcriptional, and translational processes [53]. The genes included in this category encode the CaMKII α subunit (Camk2a, 1.7-fold, RPKM = 38.4), CaMKIV (Camk4, 1.7-fold, RPKM = 15.1), and death-associated protein kinase 1 (Dapk1, 1.8-fold, RPKM = 29.7). Quantification by HCS microscopy revealed a clearly bimodal expression pattern with 45±0.7% being positive for CaMKIIα (Fig. 4G, I). Double labeling with TRPV1 showed that 56±2% of CaMKIIα(+) neurons also expressed TRPV1 and that mean signal intensities were higher in TRPV1(+) neurons (1.5-fold, p<0.05). Also the expression level of CaMKIV known to be crucial for the regulation of CRE-dependent transcription in neuronal nuclei was increased in TRPV1(+) neurons (data not shown, 2.1-fold, p<0.0001).

Next, we analyzed the expression pattern of two Kv channel-interacting proteins (KChIPs) encoded in Kcnip1 and Kcnip2, respectively. KChIP1-4 are small calcium-binding proteins important for regulating neuronal excitability by modulating the dynamic inactivation of voltage-gated Kv4 A-type potassium currents [54]. Kcnip1 (2.8-fold, RPKM = 46.5), Kcnip2 (2.1-fold, RPKM = 1.2), and Kcnip4 (1.6-fold, RPKM = 41.6), but not Kcnip3 (RPKM = 79.3) were higher expressed in TRPV1(+) neurons (Table 1). Supporting this, we obtained higher signal intensities in TRPV1(+) neurons using a monoclonal KChIP1 antibody (1.5-fold, p<0.01, Fig. 4E, H). In line with the low RPKM value of 2 for Kcnip2, we observed weak signal intensities for KChIP2 in DRG neurons (Fig. 4F). Our data therefore indicate a rather specific expression of Kcnip1 and perhaps Kcnip4 within subpopulations largely overlapping with TRPV1(+) neurons.

### PGD_2_ predominantly acts on TRPV1(+) neurons

**Figure 5:**
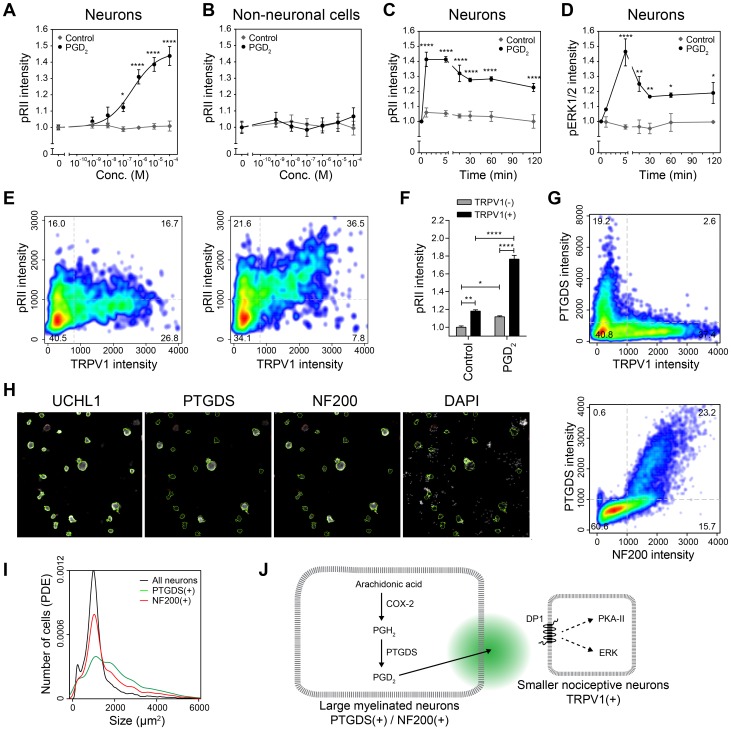
PGD_2_ is a paracrine mediator synthesized in myelinated large-diameter neurons that acts on TRPV1(+) neurons. (A) Dose-dependent induction of RII phosphorylation in sensory neurons after 1 min stimulation with PGD_2_ (EC_50_ = 377 nM, n = 3,>2000 neurons/condition; one-way ANOVA with Bonferroni's multiple comparisons test). (B) PGD_2_ did not induce pRII in non-neuronal cells of the same cultures shown in A. (C) Time course of RII phosphorylation indicating long-lasting effects of PGD_2_ (10 µM) on sensory neurons. (D) Stimulation with PGD_2_ also results in phosphorylation of the ERK1/2 measured in the same cultures shown in D. (E) Representative experiment demonstrating that induction of RII phosphorylation is enhanced in TRPV1(+) neurons (total of 3664 neurons). Plots of respective controls are shown in S2 Fig. (F) Fold changes of pRII intensities in TRPV1(−) (grey bars) and TRPV1(+) (black bars) neurons after 1 min stimulation with 10 µM PGD_2_ (n = 3,>2000 neurons/condition, one-way ANOVA with Bonferroni's multiple comparisons test). (G) Co-labeling of TRPV1 and PTGDS revealing that PTGDS is expressed in neurons lacking TRPV1 (total of 9951 neurons, also refer to S2 Fig. for control plots). (H) Co-labeling of NF200 and PTGDS showing that PTGDS(+) neurons express NF200 (total of 12966 neurons, also refer to S2 Fig. for control plots).(I) Size distribution of PTGDS(+) (green), NF200(+) (red), and all sensory neurons (black) indicating that PTGDS(+) neurons are larger than other neurons. (J) Suggested pathway of interneuronal communication between subgroups of sensory neurons. Large-diameter mechanosensitive neurons express PTGDS resulting in the production of PGD_2_, which activates DP1 receptors present on nociceptive neurons expressing TRPV1.

Next, we analyzed the functional details of one further signaling system of interest that had shown up in our transcriptome data. We found the PGD_2_ receptor DP1 (Ptgdr) to be enriched in TRPV1(+) neurons (5.0-fold, RPKM = 5.2, Table 1). To demonstrate that PGD_2_ acts on TRPV1(+) neurons, we applied an assay to monitor the activation of protein kinase A type II (PKA-II) based on measuring phosphorylation of PKA-RII subunits (pRII) recently introduced by us [39]. We observed a dose-dependent induction of RII phosphorylation after 1 min stimulation, which occurred specifically in neurons but not non-neuronal cells (Fig. 5A, B). Moreover, the response was rapid and resulted in long-lasting elevation of pRII levels above baseline (Fig. 5C), which also resulted in the transactivation of the MAPK pathway (Fig. 5D). To analyze if PGD_2_ specifically acts on TRPV1(+) neurons, we stimulated DRG neurons for 1 min with a high dose of PGD_2_ (10 µM) followed by triple staining for UCHL1, pRII, and TRPV1 (Fig. 5E). Indeed, the response to PGD_2_ was significantly higher in TRPV1(+) compared to TRPV1(−) neurons (1.8 vs. 1.1-fold, p<0.001, n = 3, total of 17970 neurons, Fig. 5F).

### PGD_2_ is synthesized in myelinated large-diameter neurons

We then aimed to determine, which cell types may be the source of PGD_2_ in sensory ganglia. The synthesis of PGD_2_ involves cyclooxygenases (COX), which are targets for non-steroidal anti-inflammatory drugs (NSAIDs) representing well-known analgesics. COX enzymes convert arachidonic acid to prostaglandin H_2_ (PGH_2_), a precursor of pain relevant prostanoids PGI_2_, PGE_2_, and PGD_2_. Interestingly, among the 87 genes showing elevated expression in cells lacking TRPV1 (<0.67-fold, p<0.05, S5 Table) such as larger myelinated neurons and glia cells, only Ptgs2 encoding COX-2 was detected by microarrays and sequencing (0.65-fold, RPKM = 11.5). In addition, also the lipocalin-type prostaglandin D synthase converting PGH_2_ to PGD_2_ was higher expressed in cells devoid of TRPV1 (Ptgds, 0.66-fold, RPKM = 66.6). To evaluate which cell types express PTGDS, we stained cultured DRG neurons with a rabbit monoclonal PTGDS antibody. To our surprise, we detected PTGDS expression in 19±2% of the investigated neurons (n = 3, total of 19769 neurons) all of which were negative for TRPV1 (Fig. 5G). PTGDS(+) neurons co-expressed the myelination marker neurofilament 200 (NF200) and were of larger size than the total neuron population (Fig. 5H, I). This suggests a paracrine circuit in which PGD_2_ is synthesized by larger myelinated neurons and activates DP1 receptors predominantly expressed by TRPV1(+) neurons (Fig. 5J).

## Discussion

With our data we present a transcriptomic approach to analyze subgroups of primary nociceptive neurons on the basis of a functional selection procedure. This resulted in the first subgroup specific transcriptome data. The differentially expressed proteins highlight that in addition to the mostly investigated ion channels and classical nociceptive markers, also signaling components are differentially expressed. Even further, the subgroup of TRPV1(+) neurons is not homogeneous but can be differentiated into further subclasses on the basis of the specific expression of signaling systems. The analysis of one such system indicates an interesting novel paracrine communication between nociceptive and non-nociceptive neurons.

The sensation of different sensory modalities is considered to reside to a large extent within distinct subgroups of specialized sensory neurons [55]. However, the identification of molecular markers and the assignment of physiological functions to subpopulations of sensory neurons remains challenging. One reason for that is the lack of methods to separate the neuronal subgroup of interest from the heterogeneous cell population within sensory ganglia. To achieve this, we established a novel agonist-based approach to selectively remove TRPV1(+) neurons from the heterogeneous cell population within sensory ganglia. Since multiple regulatory circuits control the gating and presentation of TRPV1 at the cellular surface, expression levels may not correlate with its activity. Our strategy therefore did not rely on measuring TRPV1 expression levels, but on the functional response induced by TRPV1 agonists. The method is rapid and also minimizes the influence of non-neuronal cells, which are in excess but remain unchanged in the control and agonist-treated samples.

The method allowed the identification of transcripts enriched in TRPV1(+) neurons by microarrays and sequencing based expression profiling. With the exception of two microarray based studies applying laser microdissection to compare either small- with large–diameter neurons or dorsal root and nodose ganglion neurons labeled from the peritoneal cavity [56], [57], subgroups of sensory neurons were not analyzed using whole transcriptome approaches yet. Other studies compared whole DRGs with closely related neuronal tissues such trigeminal, genticulate, or nodose ganglia to identify DRG enriched transcripts [45], [57], [58], analyzed pain relevant animal models [59], or in vitro stimulated DRG neurons [60]. RNA-Seq has been applied only recently to study gene expression in mouse trigeminal and dorsal root ganglia [45]. During the review process of this manuscript, Goswami and colleagues reported a first RNA-Seq based transcriptome of TRPV1(+) DRGs in mice and trigeminal ganglia in rats [61]. They compared FACS-enriched TRPV1(+) neurons from genetically labeled mice with DRG neurons of mice in which TPRV1(+) neurons were deleted by expressing diphtheria toxin in TRPV1(+) neurons. Transcriptome data of these experiments were compared with data of rats in which TRPV1(+) neurons were chemically ablated *in vivo* in trigeminal ganglia. Of note, although technically rather distinct, both studies identified highly similar gene sets with TRPV1-enriched expression.

Data of both studies suggest that not expression of TRPV1 alone, but also the presence of other ion channels, GPCRs, and several signaling components define the subgroup of TRPV1(+) neurons. Comparing the identified genes with known pain genes and analyzing their GO annotation verified a significant enrichment of pain-relevant genes. In addition, novel candidates were identified some of which we analyzed by us in more detail using HCS microscopy. The coexpression of components was proven to be essential for the functional outcome of nociceptive responses. In a landmark paper, Rush et al. showed that indeed the coexpressed nociceptive component NaV1.8 defines whether a single point mutation in the related NaV1.7 results in neuronal gain or loss of function [44]. Thus, our dataset will provide a rich source for the evaluation of the functional interplay of TRPV1 with other differentially expressed components.

### Ion channels enriched in TRPV1(+) neurons

Our analysis revealed multiple ion channels, especially potassium channels, being enriched in TRPV1(+) neurons (Table 1 and S5 Table). Voltage-gated potassium and sodium channels are essential for the generation of neuronal action potentials and therefore critical for the modulation of pain. For instance, we found the two-pore potassium channels TREK-1 (Kcnk2, 2.5-fold, RPKM = 11) and TRESK (Kcnk18, 1.6-fold, RPKM = 28) being differentially expressed. TREK-1 can be opened by anesthetics, is sensitive to thermal and mechanical stimulation, and was found to colocalize with TRPV1 [62], [63]. TRESK is activated by calcium involving the calcium/calmodulin-dependent protein phosphatases in cell models and has been suggested being the target of the Sichuan pepper ingredient sanshool in sensory neurons [64]. Also the tetrodotoxin-sensitive sodium channel NaV1.9 (Scn11a, 1.6-fold, RPKM = 218) is involved in the transmission of pain signals in small nonpeptidergic DRG neurons [65]. Interestingly, also gain-of-function mutations in human SCN11A result in loss of pain perception [66]. Moreover, we detected the sodium activated potassium channel Slack (Slo2.2, KCa4.1) encoded by Kcnt1 (1.7-fold, RPKM = 30). Opening of Slack channels normally dampens neuronal excitability, but internalization results in neuronal hyperexcitability [67]. Indeed, knockdown of Slack increases thermal and mechanical sensitivity in rats [68]. Also the β2 subunit of the large conductance calcium-activated potassium (BKCa) channel (Kcnmb2, 2-fold, RPKM = 8) was higher expressed in TRPV1(+) neurons. Mutant mice lacking BKCa have increased nociceptive behavior in models of persistent inflammatory pain [69].

We detected robust expression of all three α2δ family calcium channel subunits Cacna2d1-3 in sensory ganglia, but only Cacna2d1 was significantly enriched in TRPV1(+) neurons (1.6-fold, RPKM = 67.3). The ortholog of Cacna2d3 in flies was identified in a genome-wide screen for genes involved in heat nociception and also mice as well as humans mutant for Cacna2d3 show reduced sensitivity to noxious heat [48]. Our data now indicate that especially Cacnad1 may be relevant for heat pain as well. Also knock down of the potassium channel interacting proteins Kcnip1 and 4 resulted in reduced heat pain sensitivity in Drosophila [48]. We also found Kcnip1, 2, and 4 transcripts with increased expression in TRPV1(+) neurons and verified the results at protein level by HCS microscopy. The encoded proteins KChIP1-4 are calcium-binding proteins important for regulating neuronal excitability by modulating the dynamic inactivation of voltage-gated Kv4 A-type potassium currents [54]. Several studies demonstrated that KChIPs are auxiliary subunits of Kv4.1 and Kv4.3 [70], [71], which we found to be robustly expressed in DRG neurons (RPKM = 60.5 and 50.1, respectively). Kv4.3 levels were significantly increased in the TRPV1(+) population (Kcnd3, 1.4-fold). These observations may explain differences in the inactivation kinetics of potassium currents among sensory neurons. Since KChIPs bind calcium they may interlink calcium channels and other cation channels such as Trp channels with voltage gated potassium channels. Many other Trp channels such as Trpa1 (RPKM = 43), Trpc1 (18), Trpc3 (18), Trpc4 (1.4), Trpc5 (2), Trpc6 (6), Trpm2 (8), Trpm3 (34), Trpm4 (6), Trpm7 (24), Trpm8 (54), and Trpv2 (50) were found to be expressed in DRG neurons. However, only Trpc3 (2.4-fold) and Trpc4 (3.4-fold) were significantly enriched in TRPV1(+) neurons above the 1.5-fold threshold. These Trpc channels, known to be involved in the stimulus-dependent regulation of intracellular Ca^2+^ signaling, were found being relevant for light touch (Trpc3) or have been implicated with visceral pain (Trpc4) [72], [73]. In addition, we detected the α6 (Chrna6, 2.6-fold, RPKM = 49) and ε subunit (Chrne, 1.8-fold, RPKM = 21) of acetylcholine receptors enriched in TRPV1(+) neurons. These observation may be relevant to better understand the actions of nicotine on the peripheral nervous system and the analgesic actions of nicotinic acetylcholine receptor agonists such as epibatidine [74], [75]. In conclusion, the presence of multiple potassium and calcium channels as well as calcium-regulated accessory proteins suggests that TRPV1(+) neurons express a specific system to tightly control calcium triggered membrane depolarization. The enrichment of channels relevant for thermo-, mechano-, and chemosensation underlines the heterogeneous and polymodal nature of TRPV1(+) neurons.

### GPCRs enriched in TRPV1(+) neurons

We found enhanced expression of GPCRs, their ligands, and proteins relevant for GPCR signaling in TRPV1(+) neurons (Table 1 and S5 Table). These included well-known pain targets such as prostanoid, neuropeptide, and opioid receptors. We detected the galanin receptor 2 (Galr2, 2.3-fold) and its ligand galanin (Gal, 2.2-fold, RPKM = 22.6) expressed at higher levels in TRPV1(+) neurons. Galanin has been implicated with spinal nociception in several morphological, molecular, and functional studies [76]. In the periphery, galanin does not induce nociception on its own, but amplifies capsaicin induced nociceptive behaviors [77]. Our data now suggest an autocrine loop for galanin concerning the regulation of TRPV1 activity in nociceptive neurons. The two NPY receptor subtypes Npy1r (1.7-fold, RPKM = 30) and Npy5r (1.9-fold, RPKM = 2) were higher expressed in TRPV1(+) neurons. Although their ligand is not expressed in naive sensory neurons, NPY is strongly upregulated after peripheral nerve injury suggesting another autocrine loop in TRPV1(+) neurons [78], [79]. Another neuropeptide detected by us with a high-fold change is CART (11-fold, RPKM = 18). The CART mRNA encodes two peptides implicated in several physiological functions such as food intake, reward, and other endocrine functions [80]. CART has been shown to potentiate spinal N-methyl-D-aspartate (NMDA)-mediated nociceptive transmission [50], [81]. Detailed studies on spinal cord sections of mice demonstrated that ∼50% of the peptidergic nerve terminals also contain CART [51], [82]. Here we demonstrate that CART is expressed at high levels in a tiny subpopulation (≈ 3%) of sensory neurons co-expressing TRPV1. This fits to recent data on rat trigeminal ganglia in which only 1.3% of neurons expressed CART [83].

We also found the two Mas-related GPCR family members Mrgprd (4.4-fold, RPKM = 46) and Mrgprx3 (3.8-fold, RPKM = 33). Mrgprd is selectively expressed in nonpeptidergic nociceptors and was shown to respond to β-alanine [84]. In mice, Mrgprd influences the excitability of polymodal nonpeptidergic nociceptors to mechanical and thermal stimuli [85]. However, it was reported that the Mrgprd(+) population does not overlap with TRPV1(+) neurons in mice [5], which may indicate a species-specific difference between mice and rats. Recent findings indicate that members of the Mrgpr family are mediators of histamine-independent itch [86], [87], but the functions of Mrpgdx3 remained elusive.

Three GPCRs currently classified as orphan receptors were enriched in TRPV1(+) neurons. Gpr27 (1.6-fold, RPKM = 3) is conserved among mammals, was detected in grey matter areas of the monkey brain, and was recently identified as positive regulator of insulin production pancreatic beta cells [88], [89]. For Gpr35 (1.6-fold, RPKM = 20), kynurenic acid and 2-acyl lysophosphatidic acid have been proposed as endogenous ligands [90], [91]. Also pamoic acid, which is used to formulate numerous drugs, has been suggested being a Gpr35 agonist that attenuates visceral pain perception in the mice writhing test [92]. Gpr68 (OGR1, 1.7-fold, RPKM = 7) may represent an acid sensing G_q/11_-coupled GPCR in various cell types [93]. Knock-out studies in mice implicate Gpr68 with osteoclastogenesis and tumor formation [94]. Gpr68 transcripts were detected in small-diameter DRG neurons of mice [95], but its function in the context of pain is not known yet.

In addition to GPCRs and some of their ligands, we also found the regulators of G protein signaling Rgs4 (2.4-fold, RPKM = 561), Rgs7 (1.6-fold, 51.6), and Rgs14 (2.7-fold, RPKM = 1.3) enriched in TRPV1(+) neurons. Especially Rgs4 was among the most highly expressed genes with only 63 other genes showing higher RPKM values in sensory neurons. RGs proteins are considered as modulatory proteins that increase the GTPase activity of active Gα subunits resulting in rapid turnoff of GPCR signaling pathways [96]. Indeed, also the Gα subunits Gna14 (1.8-fold, RPKM = 132) and Gnaq (1.6-fold, RPKM = 152) were higher expressed in TRPV1(+) neurons suggesting tight control of GPCR mediated signaling in these neurons.

### TRPV1(+) neurons present enhanced adenosine and phospholipid metabolism

Genes relevant for several metabolic and signaling pathways were significantly enriched in TRPV1(+) neurons (Table 1 and S5 Table). For instance we noticed genes involved in adenosine metabolism such as prostate acid phosphatase (Acpp) and 5′-nucleotidase (Nt5e). These enzymes are secreted from nociceptive neurons and dephosphorylate extracellular adenosine monophosphate (AMP) to adenosine, which results in suppression of pain by activation of A1-adenosine receptors in the dorsal spinal cord [97], [98].

Recently, the construction of a global heat pain network pointed towards phospholipid signaling as a central node in pain processing [99]. The predictions of the network were tested by analyzing the phenotype of PIP5Kα and PI3Kγ mutant mice, which both presented hypersensitivity to noxious heat pain. Although we did not detect these isoforms being enriched in TRPV1(+) neurons, genes associated with phosphatidylinositol signaling were overrepresented in our dataset. G_q/11_-coupled GPCRs activate phospholipase C, which cleaves phosphatidylinositol 4,5-bisphosphate in diacylglycerol (DAG) and inositol trisphosphate (IP3). We detected G_q/11_-coupled GPCRs (e.g. Mrgprd, Mrgprdx3, Galr2) and phospholipase Cβ3 (Plcb3, 1.7-fold, RPKM = 130) with enhanced expression in TRPV1(+) neurons. Moreover, we noticed the DAG kinases eta, iota, and zeta (Dgkh, Dgki, Dgkz), which convert DAG to phosphatidic acid. Phosphatidic acid is then converted by phosphatidate cytidylyltransferase 2 also found by us to be differentially regulated (Cds2, 1.6-fold, RPKM = 364) to CDP-diacylglycerol, which is an important precursor for the biosynthesis of phosphatidylinositol.

While DAG activates proteinkinase C, IP3 induces calcium-influx from internal stores resulting in activation of Ca^2+^/Calmodulin-dependent kinases of which we found three (CaMKIIα, CaMKIV, Dapk1) in our expression analysis. CaMKIIα has been reported to be enriched in small diameter neurons including TRPV1(+) [100]–[103] and is involved in inflammatory pain responses [104], [105]. Quantifying CaMKIIα expression by HCS microscopy resulted in a clearly bimodal distribution that overlapped with TRPV1(+) neurons. We also observed faint CaMKIV immunoreactivity mainly in nuclear regions of TRPV1(+) neurons. CaMKIV is considered as a nuclear kinase that phosphorylates cyclic AMP-response element binding protein (CREB). However, the role in sensory neurons remains unclear, since CaMKIV knockout in mice does not influence behavioral responses to acute noxious stimuli or to prolonged injury [106].

### PGD_2_ is a paracrine mediator synthesized in myelinated large-diameter neurons that acts on TRPV1(+) neurons

We found receptors for the prostanoids PGE_2_, PGD_2_, and PGI_2_ with predominant expression in TRPV1(+) neurons. Prostanoids are generally considered as pronociceptive in the periphery and are well-known downstream targets of NSAIDs [107]. We have recently shown that PGI_2_ selectively acts on PKA-RIIβ(+) neurons that include TRPV1(+) neurons, whereas PGE_2_ has a broader effect [39]. This is in line with our gene expression data indicating enhanced expression of PGI_2_ receptors in TRPV1(+) neurons (Ptgir, 1.9-fold, RPKM = 32).

In contrast to the sensitizing effects of pro-inflammatory PGE_2_ and PGI_2_, data on PGD_2_ are inconsistent [108]. For instance PGD_2_ did not sensitize primary afferents to chemical and heat stimuli [109] and was shown to be anti-inflammatory [110], [111]. On the other hand, PGD_2_ depolarized axons in the vagus nerve [112], increased the CGRP release from trigeminal neurons [113], and induced tetrodotoxin-resistant Na^+^ currents in DRG neurons [108] suggesting pain sensitizing effects. Effects of PGD_2_ are mediated by receptor subtypes DP1 and DP2, which either stimulate or inhibit cAMP formation. We found increased expression of DP1 receptors in TRPV1(+) neurons (Ptgdr, 5-fold, RPKM = 5.2), whereas DP2 receptors were apparently not expressed (Ptgdr2, RPKM<0.1). Consistently, PGD_2_ stimulation of cultured DRG neurons induced PKA-RII phosphorylation predominantly in TRPV1(+) neurons, but not non-neuronal cells (Fig. 5A-F). Moreover, we detected both enzymes of the PGD_2_ biosynthesis pathway, namely COX-2 (Ptgs2, 0.65-fold, RPKM = 11.5) and prostaglandin D synthase (Ptgds, 0.66-fold, RPKM = 66.6), with reduced expression in TRPV1(+) neurons. Further analysis using a PTGDS-specific antibody revealed predominant expression in myelinated large-diameter neurons by HCS microscopy (Fig. 5G-I). These findings suggest that PGD_2_ is produced within sensory ganglia by myelinated large-diameter neurons and acts as a paracrine mediator on TRPV1(+) cells (Fig. 5J). This interneuronal communication may be of relevance within ganglia, unmyelinated areas within nerve fibers, or at nociceptor terminals. Larger unmyelinated areas that allow interneuronal communication apart from synapses have recently been reported for the central nervous system [114].

### Conclusions

In conclusion, our results demonstrate a rapid and function-driven method to remove TRPV1(+) neurons from the heterogeneous cell population within sensory ganglia. This enabled for the first time the detailed transcriptome analysis of a defined subgroup of sensory neurons. Our findings indicate that TRPV(+) neurons are not defined by ion channel alone, but are enriched for various ion channels, G-protein coupled receptors, and signaling components (e.g. adenosine and the phosphatidylinositol pathway). Indeed, the differential expression of signalling components further divides the subgroup of TRPV1(+) neurons. The analysis of one such signalling system identified PGD_2_ as a potential paracrine mediators produced in myelinated large-diameter neurons that predominately acts on TRPV1(+) neurons suggesting interneuronal communication between subgroups of sensory neurons. It remains to be analysed in detail, what differential functionality is embodied by the novel subclasses of TRPV1(+) neurons. Further, it will be of great interest to characterise the functional interplay of differentially expressed components and TRPV1.

## Materials and Methods

### Antibodies

The following antibodies were used in this study: chicken polyclonal anti-UCHL1 (1∶2000, Novus, Cambridge, UK, #NB110-58872), rabbit polyclonal anti-TRPV1 (1∶1000, Alomone labs, Jerusalem, Israel, # ACC-030), goat polyclonal anti-TRPV1 (1∶500, R&D Systems, #AF3066), mouse monoclonal anti hCART, (1∶3000, R&D Systems, #MAB163), rabbit monoclonal anti NOS1 (1∶500, clone C7D7, Cell Signaling, Danvers, MA, #4231), mouse monoclonal anti CaMKIIα (1∶2000, clone 6G9, Thermo Scientific, #MA1-048), rabbit monoclonal anti CaMKIV (1∶500, Millipore, clone EP2564Y, #04-1081), mouse monoclonal anti KChIP1 (1∶500, Abcam, Cambridge, UK, #ab99013), mouse monoclonal anti KChIP2 (1∶1000, UC Davis/NIH NeuroMab Facility, Clone K60/73, #75-004), rabbit monoclonal anti phospho RIIα (S96) (1∶1000, clone 151, Abcam, # ab32390), mouse monoclonal anti phospho-p44/42 MAPK (T202/Y204) (1∶250, clone E10, Cell Signaling, #9106), rabbit monoclonal anti-Prostaglandin D Synthase (1∶1000, clone E12357, Abcam, ab182141), mouse monoclonal anti-NF200 (1∶2000, clone N52, Sigma-Aldrich, Munich, Germany, #N0142), highly cross-adsorbed Alexa 647, 594, and 488 conjugated secondary antibodies (Invitrogen, Carlsbad, CA).

### Drugs

Capsaicin and RTX were purchased from Sigma-Aldrich (Munich, Germany) and dissolved in DMSO to 10 mM and 100 µM stocks. Prostaglandin D_2_ was purchased from Cayman (Ann Arbor, MI). All drugs were prepared as 10 or 100 mM stocks in PBS or DMSO.

### Animals

All experiments were performed with male Sprague Dawley rats (200–225 g, 8–10 weeks old) obtained from Harlan (Rossdorf, Germany). All animal experiments were performed in accordance with the German animal welfare law and approved by the Landesamt für Gesundheit und Soziales Berlin (Permit Number: ZH120). The rats were sacrificed between 9–12 a.m. by CO_2_ intoxication and L1–L6 DRGs were removed within 30 min per animal.

### DRG neuron cultures

L1–L6 DRGs were de-sheathed, pooled and incubated in NeurobasalA/B27 medium (Invitrogen, Carlsbad, CA) containing collagenase P (Roche, Penzberg, DE) (0.2 U/ml, 1 h, 37°C, 5% CO_2_). The DRGs were dissociated by trituration with fire-polished Pasteur pipettes. Axon stumps and disrupted cells were removed by BSA gradient centrifugation (15% BSA, 120 g, 8 min). Viable cells were resuspended in NeurobasalA/B27 medium, plated in poly-L-ornithin (0.1 mg/ml)/laminin (5 µg/ml)-precoated 96 well imaging plates (Greiner, Kremsmünster, AU) or onto glass cover slips (12 mm diameter), and incubated overnight (37°C, 5% CO_2_). Neuron density was 1500 neurons/cm^2^.

### Frozen DRG sections

Prepared L3–L6 DRGs were fixed with 2% paraformaldehyde for 4h on ice, rinsed 3x for 20 min with PBS at RT, and submerged in 30% sucrose in PBS at 4°C overnight. The tissues were embedded in Tissue tek (EMS Science Services) and snap frozen on dry ice. Frozen blocks were cut in 20 µm sections using a Cryostar Cryostat HM560, mounted on slides, dried for 30 min at RT, and stored at −80°C. Thawed sections were postfixed in 2% paraformaldehyde for 10 min at 4°C, rinsed in PBS for 30 min., and stained as described below. Confocal images were acquired using a Zeiss LSM 700 and processed using Image J [115].

### Depletion of TRPV1(+) DRG neurons

Lumbar L1–L6 DRGs were removed from male Sprague Dawley rats as described above and incubated in 5 ml MEM containing collagenase P (Roche, Penzberg, DE) (0.1 U/ml, 1 h, 37°C, 5% CO_2_). The DRGs were dissociated by trituration with fire-polished Pasteur pipettes. The cell suspension was divided in up to 5 aliquots, the TRPV1 agonists were added in the respective concentrations in a final volume of 5 ml per condition, and incubated for 30 min (unless otherwise stated) at 37°C. Controls were treated with the solvent DMSO up to 0.1%. The cells were spun down (100 g, 5 min), resuspended in 1 ml Hank's buffered salt solution (pH 8.0) containing 0.025% EDTA and 245 U/ml trypsin (Worthington, Lakewood, NJ), and incubated for 4 min at 37°C in a water bath. Trypsination was stopped by the addition MgSO_4_ (400 µM final conc.). The cell suspension was then loaded onto BSA gradients (15% BSA in Hank's buffered salt solution) and centrifuged (120 g, 8 min). The pellet was frozen in liquid nitrogen and stored at −80°C for RNA isolation.

### qPCR

RNA isolation was performed with NucleoSpin RNA/Protein kits from Macherey-Nagel according to the manufactures instructions including on column DNase treatment. The RNA concentration and quality was measured with spectroscopy (Nanodrop, Thermo Fisher Scientific) and capillary electrophoresis (Agilent 2100 Bioanalyzer). Approximately 1 µg total RNA was reverse transcribed using the Multi-Scribe RT kit (Applied Biosystems, Carlsbad, CA) with random hexamers. Reactions were performed in triplicate using SYBR Green I master mix (Applied Biosystems, Carlsbad, CA). Normalization and error propagation were calculated as described [116]. Relative quantities were normalized to beta-actin. Sequences of qPCR primer pairs are provided in S8 Table.

### Gene expression profiling with Illumina RatRef-12v1 arrays

DRGs isolated from one rat were split into three aliquots during the depletion procedure (see above) and treated with solvent DMSO (0.1%), capsaicin (10 µM), or RTX (100 nM) for 30 min. Four replicate experiments were performed on different days at the same day time resulting in 12 samples. RNA isolation was performed with NucleoSpin RNA/Protein kits from Macherey-Nagel including on column DNase treatment. The RNA concentration and quality was measured with spectroscopy (Nanodrop, Thermo Fisher Scientific) and capillary electrophoresis (Agilent 2100 Bioanalyzer, 28S:18S rRNA ratio>1.7, RNA integrity number (RIN)>8.2). The samples (500 ng total RNA per sample) were hybridized onto one Illumina RatRef-12 v1.0 bead chip containing 12 arrays according to the Illumina's direct hybridization protocol in our in house Illumina core facility. The microarray design is available at GEO database (GPL6101). Raw fluorescence intensities were background corrected and exported from the Illumina Genome studio software (V210.3). Background corrected data were log2 transformed, normalized (quantile method), and filtered for expressed genes (Illumina detection *p-value* <0.01 in at least one of the samples) using the R package lumi [117]. Differentially expressed transcripts were identified with the R package limma by fitting a linear model to the expression data for each probe using the least square regression method followed by an empirical Bayes method to rank genes [118]. P-values were adjusted for multiple testing with Benjamini and Hochberg's method. Microarray data are available online at the GEO database (GSE59727). The used R script and all input tables are provided in S1 Data.

### Transcriptome Sequencing (RNA-seq)

DRGs isolated from three rats were split into half and treated with solvent DMSO (0.1%) or RTX (100 nM) for 30 min. Three replicate experiments were performed at the same time on different days resulting in 6 samples. RNA isolation was performed with NucleoSpin RNA/Protein kits (Macherey-Nagel, Dueren, Germany) including on column DNase treatment. The RNA was quality controlled with capillary electrophoresis (Agilent 2100 Bioanalyzer, 28S:18S rRNA ratio>1.7, RNA integrity number (RIN) between 7.1 and 8.3). Ribosomal RNA was removed from 5 µg total RNA per sample using Ribo-Zero rRNA Removal Kit (Epicentre Biotechnologies, Madison, WI, USA). Sequencing libraries were prepared using SOLiD Total RNA-Seq Kit (Life Technologies, Carlsbad, CA, USA) according to the manufacturer's instructions. Samples were sequenced as a pool of barcoded samples on three lanes of an ABI 5500xl flowchip. The yield was between 73 million and 174 million of 75 bp and 35 bp long reads per sample (664 million reads in total). SOLiD 5500xl produces XSQ files, containing the reads in a binary format, which were used as input for Lifescope-v2.5.1-r0 [http://www.lifetechnologies.com/lifescope]. Lifescope is used for aligning the reads to the reference genome (rn5). In addition to the reads in the XSQ files, three reference input files are used: filter reference, genome reference and annotation file. The filter reference is a fasta file containing ribosomal rna, tRNA and vector sequences and is used in the first step to exclude reads aligning to these sequences. The genome reference is used in the second step to align the remaining reads to the complete reference genome. The aligned reads are then used in the third step in conjunction with the annotation file [http://hgdownload.cse.ucsc.edu/goldenPath/rn5/database/refGene.txt.gz, accessed 2012-11-26] to compute the number of reads that align within exons of the transcriptome. Normalization of read counts to RPKM values was done by Lifescope. Loess normalization of read counts by GC content and full quantile normalization of read counts was done using the R package EDASeq [119]. The function ‘nbinomTest’ of the package DESeq [120] was used to compute the Benjamini-Hochberg adjusted p values for difference between the base means. Raw RNA-Seq data are available online at the ArrayExpress (E-MTAB-2789). The reference transcriptome, the R script for analysis, and all input tables are provided in S2 Data.

### Western blotting

L1–L6 DRGs were pulverized in liquid nitrogen and lysed in 1 ml lysis buffer (15 mM Tris/HCl (pH 7.5), 8 M urea, 8.7% glycerol, 1% sodium dodecyl sulfate, 143 mM β-mercaptoethanol). Lysates were homogenized (QIAShredder, Qiagen, Hilden, DE), denatured for 5 min at 95°C, loaded (10 µg), separated by SDS-PAGE, and transferred to PVDF membranes (Immobilon-P; Millipore, Billerica, MA). After blocking in Tris buffered saline (TBST) with 2.5% milk powder at 4°C overnight, membranes were incubated with the primary antibody diluted in TBST for 3 h at RT. After three washes with TBST (10 min, RT), the detection was performed with a chemiluminescence detection system (Thermo Fisher, Rockford, IL).

### Immunofluorescence staining

After blocking and permeabilization (2% goat serum, 1% BSA, 0.1% Triton X-100, 0.05% Tween 20, 1 h, RT) of PFA-fixed sections or cells, the cultures were incubated with the respective primary antibodies diluted in 1% BSA in PBS at 4°C overnight. Subsequent to three washes with PBS (10 min, RT) cells were incubated with secondary Alexa dye-coupled antibodies (1∶1000, 1 h, RT). After three final washes (10 min, RT), wells of 96 well plates were filled with PBS, sealed, and stored at 4°C until scanning. DRG sections were mounted with Fluoromount-G (Southern Biotech, Birmingham, AL) onto slides.

### Quantitative microscopy

Stained cultures in 96-well plates were scanned using a Cellomics ArrayScan VTI. Images of 512×512 pixels were acquired with a 10x objective and analyzed using the Cellomics software package. Briefly, images of UCHL1 stainings were background corrected (low pass filtration), converted to binary image masks (fixed threshold), segmented (geometric method), and neurons were identified by the object selection parameters: size: 120–4000 µm^2^, circularity (perimeter2/4π area): 1–2, length-to-width ratio: 1–2, average intensity: 250–2000, total intensity: 6×10^4^−5×10^6^. The image masks were then used to quantify signals in other channels. Three respective controls were prepared for each triple staining: (1) UCHL1 alone, (2) UCHL1 + antibody 1, and (3) UCHL1 + antibody 2. Raw fluorescence data of the controls were used to calculate the spill-over between fluorescence channels. The slope of best fit straight lines were determined by linear regression and used to compensate spill-over as described (Roederer, 2002). Compensated data were scaled to a mean value of 1 (or 1000) for the unstimulated cells to adjust for variability between experimental days. One and two-dimensional probability density plots were generated using R packages [121]. Gating of subpopulations was performed by setting thresholds at local minima of probability density plots.

### Statistical Analysis

Statistical analyses were performed with paired two-tailed t-tests or One-way ANOVA as indicated in the respective figure legend. P<0.05 was considered as statistically significant. To determine whether differentially expressed genes are enriched for pain relevant genes listed in databases, we used the Fisher's exact test implemented in R with the assumption that the alternative hypothesis must be ‘greater’ indicating overrepresentation in the dataset. We assumed a total number of 22777 coding genes in the rat genome.

## Supporting Information

S1 Fig **Control plots for each triple staining shown in** **Fig. 4** **.** Three respective controls were prepared for each triple staining: (1) UCHL1 alone, (2) UCHL1 + antibody 1, and (3) UCHL1 + antibody 2. Raw fluorescence data of the controls were used to calculate the spill-over between fluorescence channels by linear regression (see materials and methods). The plots show data after compensation of spill-over. Data points aligned with the x- or y-axis in the middle plots indicate proper compensation of spill over.
(TIF)

S2 Fig **Control plots for each triple staining shown in** **Fig. 5** **.** Three respective controls were prepared for each triple staining: (1) UCHL1 alone, (2) UCHL1 + antibody 1, and (3) UCHL1 + antibody 2. Raw fluorescence data of the controls were used to calculate the spill-over between fluorescence channels by linear regression (see materials and methods). The plots show data after compensation of spill-over. Data points aligned with the x- or y-axis in the middle plots indicate proper compensation of spill over.
(TIF)

S1 Table **All genes detected in sensory ganglia with a detection p-value <0.01 using microarrays.**
(XLSX)

S2 Table **Genes identified with differential expression using microarrays after treatment with capsaicin (402 genes) or RTX (1015 genes), respectively (Benjamini and Hochberg adjusted p-value <0.05).**
(XLSX)

S3 Table **All genes detected with RNA-Seq in sensory ganglia (>0.1 RPKM, 13095 genes).**
(XLSX)

S4 Table **Genes identified with differential expression using RNA-Seq after removal of TRPV1(+) neurons with RTX treatment (Benjamini and Hochberg adjusted p-value <0.05).**
(XLSX)

S5 Table **Merged data of differentially expressed genes detected by microarrays and sequencing (adjusted p-values <0.05 and fold-changes >1.5 using one of the two approaches).**
(XLSX)

S6 Table **Overlap of genes detected by with higher expression levels within the TRPV1(+) subgroup and databases such as the Pain Networks database** [35] **and Pain Genes database** [36] **as well as with a genome-wide Drosophila screen for genes involved in heat nociception** [37] **.**
(XLSX)

S7 Table **Overrepresented Gene Ontology (GO) terms, Kegg pathways, and Interpro protein domains with the TRPV1-associated genes detected using microarrays and/or RNA-Seq.** The analysis was performed with DAVID [38].
(XLSX)

S8 Table **Sequences of qPCR primer pairs.**
(XLSX)

S1 Data **Supplemental information for the microarray analysis including the used R script and all input tables.**
(ZIP)

S2 Data **Supplemental information for RNA-Seq including the used R script and reference transcriptome.**
(ZIP)
