# Supplementary figures and images for: Subgroup-Elimination Transcriptomics Identifies Signaling Proteins that Define Subclasses of TRPV1-Positive Neurons and a Novel Paracrine Circuit
Source: PLoS One. 2014 Dec 31;9(12):e115731. doi: 10.1371/journal.pone.0115731 (PMC4281118; doi:10.1371/journal.pone.0115731)

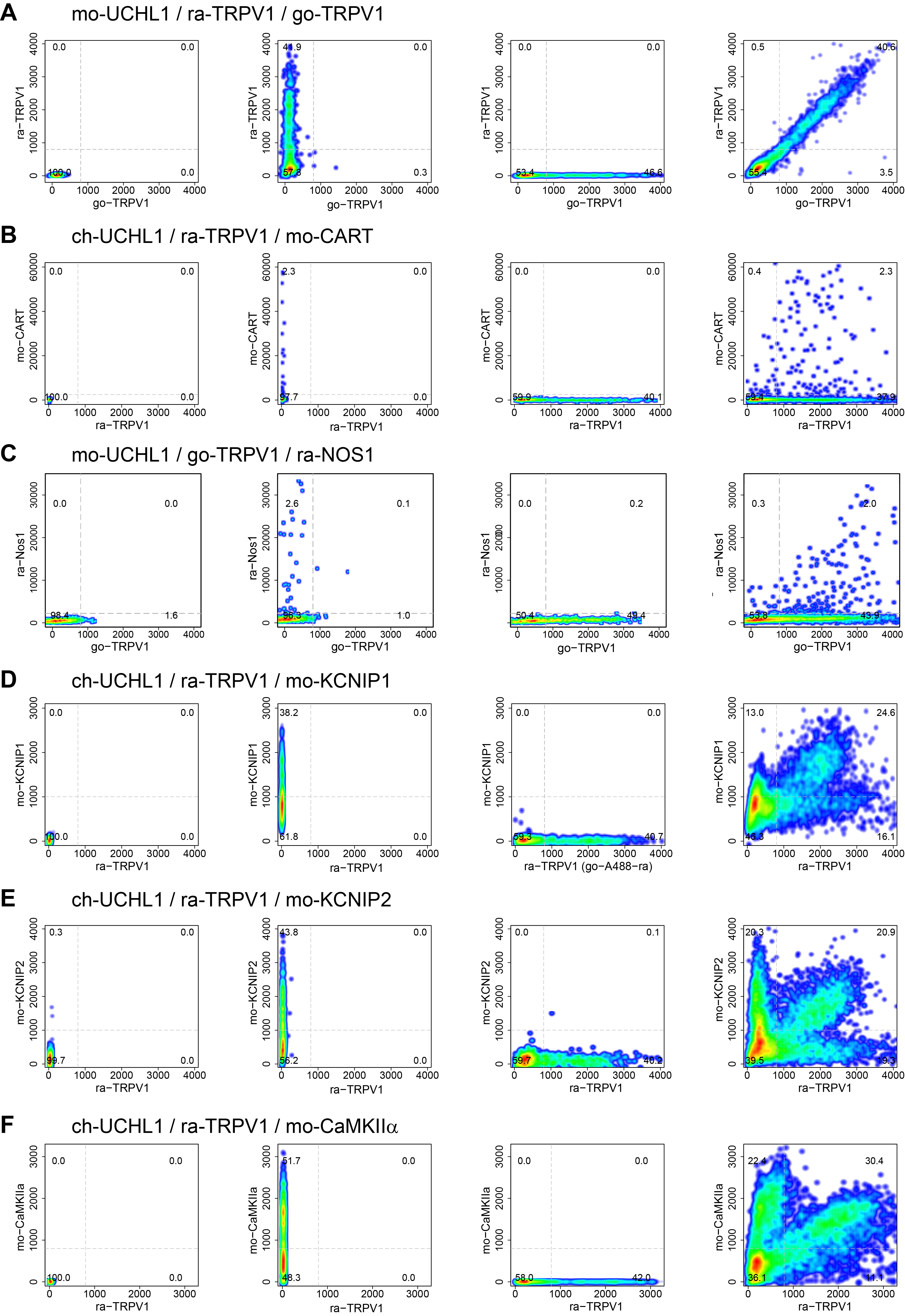

Supplement: S1 Fig — Control plots for each triple staining shown in Fig. 4 . Three respective controls were prepared for each triple staining: (1) UCHL1 alone, (2) UCHL1 + antibody 1, and (3) UCHL1 + antibody 2. Raw fluorescence data of the controls were used to calculate the spill-over between fluorescence channels by linear regression (see materials and methods). The plots show data after compensation of spill-over. Data points aligned with the x- or y-axis in the middle plots indicate proper compensation of spill over. (TIF) [file pone.0115731.s001.tif]

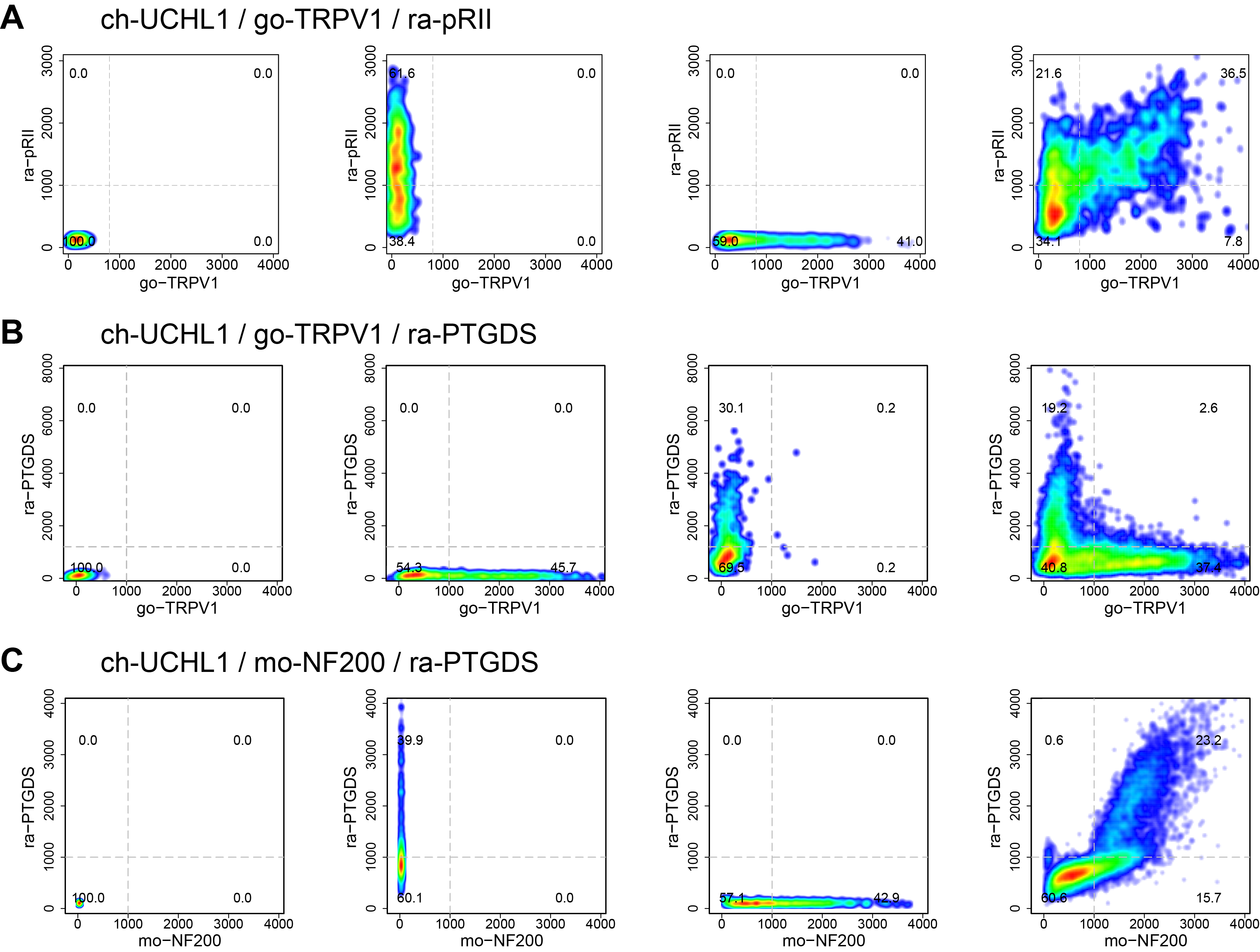

Supplement: S2 Fig — Control plots for each triple staining shown in Fig. 5 . Three respective controls were prepared for each triple staining: (1) UCHL1 alone, (2) UCHL1 + antibody 1, and (3) UCHL1 + antibody 2. Raw fluorescence data of the controls were used to calculate the spill-over between fluorescence channels by linear regression (see materials and methods). The plots show data after compensation of spill-over. Data points aligned with the x- or y-axis in the middle plots indicate proper compensation of spill over. (TIF) [file pone.0115731.s002.tif]
